# Health literacy, associated lifestyle and demographic factors in adult population of an English city: a cross‐sectional survey

**DOI:** 10.1111/hex.12440

**Published:** 2016-01-15

**Authors:** Joanne Protheroe, Rebecca Whittle, Bernadette Bartlam, Emee Vida Estacio, Linda Clark, Judith Kurth

**Affiliations:** ^1^ Research Institute for Primary Care and Health SciencesKeele UniversityKeeleUK; ^2^ School of PsychologyKeele UniversityKeeleUK; ^3^ Public Health DirectorateStoke‐on‐Trent City CouncilStoke‐on‐TrentUK

**Keywords:** health literacy, inequalities, public health

## Abstract

**Background:**

Lower health literacy is a public health issue that follows a social gradient, potentially reinforcing existing health inequalities. However, levels of health literacy in particular populations can be unclear and are a key to identifying effective public health interventions. This research examined health literacy levels in Stoke‐on‐Trent, where 31.2% of the population live in areas classified amongst the 10% most deprived in England.

**Methods:**

A cross‐sectional survey using the Newest Vital Sign examined associations with demographic factors, lifestyle behaviours, Internet use and self‐rated health. The sample (*n* = 1046) took account of variance in levels of health literacy by age, educational attainment and deprivation. Bivariate logistic regression and multivariate logistic regression were used to estimate associations with health literacy when adjusted for other demographic factors and lifestyle behaviours.

**Results:**

Nine hundred and seventy‐two respondents completed the health literacy measure (93%): 277 (28.5%) scored low, 228 (23.5%) scored marginal and 467 (48.0%) scored adequate. Associations with higher rates of limited health literacy included older age, lower educational level, lower income, perceived poor health and lack of access to the Internet.

**Conclusions:**

Given the complexity of factors influencing health literacy interdisciplinary approaches across health and social care and the voluntary sector are essential in identifying and developing appropriate interventions.

## Background

Links between health literacy (defined as ‘The degree to which individuals have the capacity to obtain, process and understand basic health information and services needed to make appropriate health decisions’) and health status is well documented.^1^ In 2011, a systematic review examining the role of health literacy on interventions and outcomes found a wide range of research that identified consistent associations between health literacy level and hospitalizations, greater use of emergency care, poorer ability to demonstrate taking medications appropriately, poorer ability to interpret labels and health messages, and poorer overall health status and higher mortality.^2^

Studies have shown a strong association between hospitalization in emergency department populations and patient health literacy; patients with inadequate literacy are twice as likely to be hospitalized when compared to individuals with adequate literacy.^3^, ^4^ In addition, studies from the US and the UK have shown that limited health literacy in the elderly population is independently associated with increased mortality.^5^, ^6^, ^7^ Lower health literacy is a public health issue which has been shown to follow a social gradient, potentially reinforcing existing health inequalities.^8^

Whilst the links between health literacy and health outcomes are well recognized, much less is known about the development of successful public health interventions to address these issues. Indeed, public health campaigns to improve health between 2003 and 2008 were more successful in the more highly educated sector of the population.^9^ People with no educational qualifications were five times more likely to partake in unhealthy behaviours increasing their likelihood to have poorer health outcomes, thus widening the divide between the least and most educated sectors of the population.^9^

In 2012, Keele University undertook a pilot study, on behalf of Stoke‐on‐Trent City Council, on the health literacy needs of men with diabetes.^10^ As a result, it was recognized that insufficient data existed about population‐level health literacy levels across the city. This paper describes the consequent cross‐sectional survey begun in 2013 to provide such data to increase understanding of the scale of the challenge. By examining associations with low health literacy, findings will inform the design of future interventions to reduce health inequalities and improve public health in the city.

The survey used a measure of functional health literacy, the ‘Newest Vital Sign’ (NVS), validated for use in a UK population but to our knowledge not used across a city‐wide population in the UK before.^11^ We investigated the prevalence of health literacy and examined associations with demographic factors, lifestyle behaviours, including Internet use and social inclusion (linked to increased health literacy in previous studies^12^), and self‐rated health.

## Methods

### Participants

The survey sample was designed to take account of variance in levels of health literacy by age, educational attainment and deprivation. Overall, in the city, 31.2% of the population live in areas classified amongst the 10% most deprived in England, and the proportion varies significantly across the different geographical areas within the city. The sampling scheme was designed to obtain a sample of respondents from all the different areas in the city with quotas set on age and gender. Data collection was funded by Stoke‐on‐Trent City Council and was carried out by a market research company. Households from each enumeration district were sampled at random, achieving a sample of 1301 adult respondents; of these, 1046 respondents agreed for their data to be shared with the research team. The project was reviewed by the Keele University Ethics Research Panel and was approved prior to participant recruitment.

### Procedure

The survey was carried out face‐to‐face in respondent's homes and was designed to be completed in <15 min. The survey consisted of questions to determine demographics, self‐rated health, measure of social connectedness [Health Education Monitoring Survey (HEMS)],^13^ self‐rated lifestyle, Internet access^14^ and health literacy as measured by the NVS.^11^

The NVS was developed in the US and is a validated predictor of functional health literacy; as previously mentioned, the version used in this study was validated for use in a UK population. Unlike other health literacy measures that are self‐reported, this study used the NVS to enable participants to demonstrate actual functional health literacy skills. It takes approximately 3 min to administer and consists of a food nutrition label (similar to one to be found on an ice cream container) with six associated questions measuring both literacy and numeracy skills. Respondents receive one point for each correct answer. A score of 0–1 indicates a high likelihood that the patient has limited literacy. A score of 2–3 indicates a possibility of limited literacy, and a score of 4–6 almost always indicates a patient has adequate literacy to navigate the health‐care system. It is reliable and acceptable to patients and correlates well with the much lengthier Test of Functional Health Literacy (TOFHLA), commonly used in testing health literacy levels.^11^

The measure of social connectedness used was from the HEMS by the Social Survey Division of the Office for National Statistics 1998 and consisted of two short questions: ‘Do you have any close relatives whom you speak to or see regularly?’ and ‘Do you have any close friends whom you speak to or see regularly?’ Respondents were asked whether they considered themselves to have a healthy lifestyle, on a 5‐point scale from ‘very healthy lifestyle’ to ‘very unhealthy lifestyle’, whether and where they had access to the Internet and whether they used the Internet for medical, or health‐related, information.

### Statistical analysis

We used Stata/MP 13.1 (Stata Corporation, College Station, TX, USA) for data analysis.

For the descriptive analysis, as recommended in the paper validating the use of the NVS for UK populations,^11^ we described respondents who scored 0–1 on the NVS as having low functional health literacy, 2–3 as having marginal health literacy and a score of 4–6 as adequate. We combined the low and marginal categories into one category of limited health literacy for the bivariate and multivariable analyses.

Bivariate logistic regression and multivariable logistic regression were used to determine the associations between health literacy, measured by the NVS, and their characteristics (age, gender, ethnicity, education, income, index of multiple deprivation, perceived health, perceived lifestyle, social isolation and access to the Internet).

Bivariate logistic regression was performed for each of the factors, and those that were statistically significant within the bivariate models (*P* < 0.05) were then simultaneously included in a multivariable logistic regression model to estimate the associations with health literacy when adjusted for other demographic factors and lifestyle behaviours.

## Results

### Demographic variables

From the sample of 1046 respondents, 972 (93%) completed the measure of health literacy, the NVS, and were included in the analysis. The demographics were broadly representative of the population of Stoke‐on‐Trent with slightly fewer male participants at 45.8 male and 54.2% female (adult population of Stoke‐on‐Trent is 49.5 and 50.5% respectively). The study participants were slightly older than the Stoke‐on‐Trent population with 27.5% aged 18–34 (vs. 31.2%), 47.0% aged 35–64 (vs. 47.8%) and 25.5% aged over 65 years (vs. 21.0%).

Table 1 shows the distribution of demographic variables.

**Table 1:** Relationship between respondent characteristics and health literacy levels using Newest Vital Signs (NVS), Stoke‐on‐Trent, UK, 2013

	Total	Health literacy level		
		Low	Marginal	High
Respondents	972 (100)	277 (28.5)	228 (23.5)	467 (48.0)
Age; mean (SD)	48.7 (18.8)	59.0 (18.9)	49.0 (17.9)	42.4 (16.3)
Age				
18–34	266 (27.5)	38 (13.9)	57 (25.0)	171 (36.7)
35–64	454 (47.0)	100 (36.6)	118 (51.8)	236 (50.6)
65+	247 (25.5)	135 (49.5)	53 (23.3)	59 (12.7)
Male	444 (45.8)	121 (43.8)	101 (44.3)	222 (47.6)
Ethnicity				
White British	884 (91.0)	243 (87.7)	213 (93.4)	428 (91.7)
Other	88 (9.1)	34 (12.3)	15 (6.6)	39 (8.4)
Education				
None	290 (30.1)	154 (56.2)	68 (29.8)	68 (14.8)
GCSE or equivalent^a^	314 (32.6)	75 (27.4)	90 (39.5)	149 (32.3)
A‐Levels or equivalent^b^	109 (11.3)	10 (3.7)	25 (11.0)	74 (16.1)
Beyond A‐level	250 (26.0)	35 (12.8)	45 (19.7)	170 (36.9)
Household income				
<£10 000	195 (20.2)	73 (26.7)	45 (19.8)	77 (16.5)
£10 000–£19 999	181 (18.7)	43 (15.8)	43 (18.9)	95 (20.4)
≥£20 000	230 (23.8)	18 (6.6)	58 (25.6)	154 (33.1)
Don't know or prefer not to say	360 (37.3)	139 (50.9)	81 (35.7)	140 (30.0)
Deprivation (national IMD)				
Most deprived	489 (50.3)	151 (54.5)	117 (51.3)	221 (47.3)
2nd most deprived	218 (22.4)	65 (23.5)	54 (23.7)	99 (21.2)
3rd most deprived	154 (15.8)	35 (12.6)	30 (13.2)	89 (19.1)
4th most deprived	67 (6.9)	19 (6.9)	19 (8.3)	29 (6.2)
Least deprived	44 (4.5)	7 (2.5)	8 (3.5)	29 (6.2)
Perceived health				
Very good	236 (24.3)	44 (15.9)	57 (25.0)	135 (28.9)
Good	437 (45.0)	104 (37.6)	96 (42.1)	237 (50.8)
Fair	205 (21.1)	79 (28.5)	50 (21.9)	76 (16.3)
Bad/very bad	94 (9.7)	50 (18.1)	25 (11.0)	19 (4.1)
Perceived lifestyle				
Very healthy	223 (23.0)	76 (27.4)	45 (19.8)	102 (21.9)
Fairly healthy	521 (53.7)	136 (49.1)	140 (61.7)	245 (52.6)
Neither good nor bad	168 (17.3)	44 (15.9)	34 (15.0)	90 (19.3)
Fairly/very unhealthy	58 (6.0)	21 (7.6)	8 (3.5)	29 (6.2)
Social isolation				
See/speak to close friends/family	900 (92.8)	248 (90.2)	219 (96.1)	433 (92.7)
Don't see/speak to close friends/family	70 (7.2)	27 (9.8)	9 (4.0)	34 (7.3)
Internet access	755 (78.0)	147 (53.5)	176 (77.9)	432 (92.5)

Values are number (percentage) unless otherwise stated. Numbers may not add up to total due to missing data.

a General Certificate of Secondary Education (GCSE) is a single‐subject exam taken after 2 years of study at the age of 16 (age at US 10th grade).

b A levels are qualifications offered by schools and colleges for 16‐ to 19‐year‐olds.

The majority of the respondents were white, female, aged between 35 and 64 years, educated to GCSE level or less, and (amongst those prepared to state their income) currently earning £20 000 or less. Over 30% perceived their general health to be fair or poor, but over 76% perceived their lifestyle to be very or fairly healthy.

Of the 972 respondents, 277 (28.5%) had low functional health literacy, another 228 (23.5%) had marginal health literacy and 467 (48.0%) had adequate health literacy.

### Statistical analyses

Several socio‐demographic characteristics were associated with health literacy level and are shown in Table 2. Characteristics associated with higher rates of limited functional health literacy included older age, lower educational level achieved, lower income, living in a more deprived area, perceived poor health and lack of access to the Internet.

**Table 2:** Odds ratios (95% confidence intervals) of having limited (low or marginal) health literacy vs. adequate health literacy, Stoke‐on‐Trent, UK, 2013

	Bivariate analysis	Multivariable analysis
Age, years	1.04 (1.03, 1.05)***	–
Age		
18–34	1.00	1.00
35–64	1.66 (1.22, 2.27)**	1.39 (0.98, 1.97)
65+	5.74 (3.90, 8.43)***	2.48 (1.54, 4.02)***
Male	0.87 (0.67, 1.11)	–
Ethnicity		
White British	1.00	–
Other	1.18 (0.76, 1.83)	–
Education		
None	6.94 (4.74, 10.14)***	3.13 (2.04, 4.81)***
GCSE or equivalent	2.35 (1.67, 3.33)***	1.90 (1.31, 2.77)**
A‐Levels or equivalent	1.01 (0.62, 1.63)	0.91 (0.54, 1.54)
Beyond A‐level	1.00	1.00
Household income		
<£10 000	3.11 (2.09, 4.62)***	1.33 (0.84, 2.12)
£10 000–£19 999	1.83 (1.23, 2.74)**	1.00 (0.63, 1.57)
≥£20 000	1.00	1.00
Don't know or prefer not to say	3.18 (2.25, 4.50)***	1.58 (1.06, 2.35)*
Deprivation (national IMD)		
Most deprived	2.34 (1.23, 4.48)*	2.09 (0.99, 4.41)
2nd most deprived	2.32 (1.18, 4.58)*	2.07 (0.95, 4.49)
3rd most deprived	1.41 (0.70, 2.85)	1.30 (0.59, 2.90)
4th most deprived	2.53 (1.15, 5.57)*	2.30 (0.94, 5.61)
Least deprived	1.00	1.00
Perceived health		
Very good	1.00	1.00
Good	1.13 (0.82, 1.55)	0.91 (0.64, 1.30)
Fair	2.27 (1.55, 3.33)***	1.19 (0.76, 1.85)
Bad/very bad	5.28 (3.00, 9.29)***	2.27 (1.21, 4.28)*
Perceived lifestyle		
Very healthy	1.00	–
Fairly healthy	0.95 (0.69, 1.30)	–
Neither good nor bad	0.73 (0.49, 1.09)	–
Fairly/very unhealthy	0.84 (0.47, 1.50)	–
Social isolation		
See/speak to close friends/family	1.00	–
Don't see/speak to close friends/family	0.98 (0.60, 1.60)	–
No Internet access	6.80 (4.61, 10.05)***	2.80 (1.77, 4.43)***

***P < 0.001; **P < 0.01; *P < 0.05.

In the multivariable analysis, several characteristics remained significantly associated with limited health literacy, even after adjusting for all the other factors in the model. Respondents in the over 65 years age group were two and a half times more likely to have limited functional health literacy than those aged between 18 and 34 years (adjusted OR 2.48, 95% CI 1.54, 4.02), and those with no formal education were three times more likely to have limited health literacy than those with formal qualifications beyond A‐level (adjusted OR 3.13; 95% CI 2.04, 4.81).

Individuals who rated their health as bad or very bad were twice as likely to have limited health literacy compared to those who rated their health as good or very good (adjusted OR 2.27; 1.21, 4.28), and individuals who had no access to the Internet were nearly 3 times more likely to have limited health literacy than those who had access (adjusted OR 2.80; 1.77, 4.43).

Once income was taken into account in the analysis, those living in the most deprived areas were more than twice as likely to have limited health literacy than those living in the least deprived areas, although significance was borderline (adjusted OR 2.09; 0.99, 4.41).

There was, however, no significant difference in terms of social isolation (as described by seeing or speaking to friends and/or relative regularly) or perceived healthy lifestyle between those with limited health literacy and those with adequate health literacy in the bivariate analyses, and hence, these were not included in the multivariable model.

## Discussion

### Main findings

More than half of the eligible respondents in this survey (52%) were assessed from their NVS scores as having limited health literacy. Factors associated with limited functional health literacy were older age, lower formal educational level, lower income, perceived poor health and lack of access to the Internet. There was no significant association with limited functional health literacy and gender, social isolation or perceived healthy lifestyle.

### What is already known on this topic?

Our finding of 52% (28.5% low and 23.5% marginal) limited health literacy is higher than the overall figures for the NVS in the European Health Literacy Survey (EHLS) which was 45% (21% low and 24% marginal).^14^ Health literacy levels in Stoke‐on‐Trent are similar to those of some of the poorer countries in Europe – such as Bulgaria, where 29 and 25%, respectively, had low or marginal health literacy. There is no UK‐ or England‐wide figure for the NVS, but a recent study examining the mismatch between the skills of the English working‐age population and available health materials suggested that 43% of 16‐ to 65‐year‐olds would have difficulty with written health material, rising to 61% if the health material contained numerical information.^15^ As the NVS contains both textual and numerical information, perhaps the percentage with limited health literacy in this study is less surprising. Additionally, data from Public Health England (www.healthprofiles.info) in 2014 showed that health, level of deprivation and educational attainment of people in Stoke‐on‐Trent are lower than those in the England average.

Our finding that respondents over the age of 65 years are more likely to have limited health literacy is well supported in the current literature and often attributed to decline in cognitive function.^16^ However, more nuanced research is needed to explore this from a life‐course perspective, taking into account the diverse range of factors that structure our capacity to age well, for example, socio‐economic status, gender and ethnicity, and how these change over time.^17^

There is also evidence indicating that consistent Internet use may help older adults to maintain their health literacy.^18^ Our study has shown that individuals who had no access to the Internet were nearly three times more likely to have limited health literacy than those who had access. Cross‐tabulation of the data shows that, amongst the respondents, younger people were more likely to have access to the Internet. This important finding, in line with the current drive towards a ‘digital society’,^19^ has been reflected in Stoke‐on‐Trent City Council's Health Literacy Strategy where public health interventions are being developed to link to the city's move towards digital inclusion, especially in trying to improve Internet access and use amongst the older population.

### What this study adds?

This study was undertaken with the specific purpose of informing the development of public health interventions for health literacy in this locality. The results of this survey have been shared with stakeholders across the health and social care sector, third sector, education, voluntary and patient communities. It has become clear that training around health literacy awareness and the training of patient facing staff are key priorities for local people. Also, a strong preference for local voices and local input has emerged.

Accordingly, as a direct result of this survey, health literacy awareness training is being considered for incorporation into existing interventions, as well as commissioning new training courses for patient facing staff. Some areas of work that are at the early stage of progress are as follows: working with children in schools to become ‘Sports Leaders’ and ‘Playground Leaders’ (children aged between 12–16 years and 8–11 years) encouraging healthy exercise; working on a programme of training activities that will raise awareness of health literacy in patient facing staff, which will potentially include a video of local people, as local voices have been identified as key to success; and a pharmacy pilot with engaged pharmacies within the city focusing on good communication around medicines management using the ‘Teach back’ method, planned for late summer 2015. ‘Teach back’ is a health communication strategy whereby health professionals confirm that the patient understood the information by asking them to repeat or demonstrate what they have been told.

Other new interventions include improving access to the Internet and improving collaboration across the various stakeholder groups. Considering the vast amount of health information now available on the Internet, it is evident why increased access to the Internet, especially for older adults, is crucial in this digital age. However, the ability of people to appraise that information and translate it into actions for their own health is also important which is why planned interventions include access to on‐going learning, including Internet access, for example through workplaces, libraries and community centres. The importance of closer collaboration across the various voluntary, health and social care stakeholders is apparent, in particular to improve communication with the general public. Consequently, in addition to health organizations, community organizations that have expressed an interest in health literacy are being offered the opportunity to liaise with city council communications staff to improve, for example, the readability of public notices such as health leaflets, information boards and signage. Building on real, locally relevant evidence has provided a key impetus to multidisciplinary, multisector collaborations which will result in directly relevant important interventions to improve the public health of this city. This method of taking evidence into practice would be transferrable to other areas within the UK and beyond.

### Strengths and limitations

This is the first city‐wide survey of health literacy levels conducted in the UK; the new knowledge gained from it has been mapped to forthcoming development plans for public health interventions. We used the UK‐validated NVS, which has good face validity with participants due to its obvious links to a healthy diet (food labels) and correlates well with the more widely used TOFHLA. It should be noted however that the NVS only considers functional health literacy skills and not the other types of health literacy (i.e. interactive and critical). Other forms of health literacy can therefore be considered in future research. Furthermore, this study also used self‐reported health questions which may be subject to social desirability issues.

The survey was conducted face‐to‐face and reached a sample generally representative of the city population. Recruiting using a market research company did not allow us to monitor response rates, and we had a slightly older study population than that in the city, which may have resulted in a slight overrepresentation of the true extent of limited health literacy, as it is well documented that levels of health literacy decline with older age.^20^ The cross‐sectional design of this study does not allow for conclusions to be drawn about the nature of the associations with limited health literacy, and, due to the time constraints and face‐to‐face design of this study, many of the factors, such as perceived health or perceived lifestyle, were self‐reported, which may limit the clinical significance of the results. Perceived health has been used in other studies and found to correlate extremely well with actual health.^21^ However, it is of note that a high proportion (over 76%) of the study participants who describe their lifestyle as very or fairly healthy would be likely, according to the NVS results in this survey, to have significant difficulty in interpreting food labels when shopping. This suggests that this self‐reported measure should be interpreted with a degree of caution and used as part of an holistic assessment that takes into account the complex array of psycho‐social factors that are known to structure health literacy, including individual coping strategies.^22^

## Funding

This work was supported by funds from Stoke City Council.

## Conflict of interests

The authors have no conflict of interests to declare.
